# Peripartum cardiomyopathy: A case report of decompensated heart failure in a hypertensive patient

**DOI:** 10.1097/MD.0000000000037600

**Published:** 2024-03-29

**Authors:** Chukwuka Elendu, Osinachi K. Okoye

**Affiliations:** a Department of Internal medicine, University of California, Santa Cruz, CA; b Department of Internal medicine, Chukwuemeka Odumegwu Ojukwu University Teaching Hospital, Awka, Nigeria.

**Keywords:** decompensated heart failure, hypertension, medication management, peripartum cardiomyopathy, preeclampsia history

## Abstract

**Rationale:**

Peripartum cardiomyopathy (PPCM) occurring in the context of hypertension presents a unique clinical challenge. This case contributes to the medical literature by highlighting the complexities of managing heart failure in postpartum women with pre-existing hypertensive disorders, particularly when complicated by a history of preeclampsia.

**Patient Concerns:**

Mrs. O.O., a 34-year-old hypertensive woman, presented with progressive dyspnea, bilateral leg swelling, and orthopnea. Notably, she had a history of previous preeclampsia and exhibited worsening symptoms over several months.

**Diagnoses:**

The patient was diagnosed with decompensated heart failure secondary to PPCM, exacerbated by hypertension and anemia.

**Interventions:**

Therapeutic interventions included diuretics, angiotensin receptor-neprilysin inhibitors, digoxin, and anticoagulation. Additionally, lifestyle modifications and dietary restrictions were implemented.

**Outcomes:**

Following treatment adjustments, the patient demonstrated significant improvement in symptoms, exercise tolerance, and cardiac function. The transition from NYHA class III to class II heart failure indicated successful management.

**Lessons:**

This case underscores the importance of a comprehensive approach to managing PPCM in hypertensive patients, with attention to cardiovascular and obstetric factors. It highlights the effectiveness of multidisciplinary care in achieving positive outcomes and emphasizes the need for heightened vigilance in postpartum women with cardiovascular risk factors.

## 1. Introduction

Peripartum cardiomyopathy (PPCM) is a rare but severe condition characterized by heart failure occurring during the last month of pregnancy or within 5 months of giving birth. While the exact etiology remains elusive, it is postulated to involve a combination of genetic predisposition, hormonal changes, and inflammatory processes.^[1]^ Hypertensive disorders of pregnancy, such as preeclampsia, further complicate the cardiovascular landscape.^[2]^

This case report explores the distinctive presentation of decompensated heart failure in Mrs. O.K.O., a 34-year-old hypertensive woman with a history of preeclampsia. The literature highlights the increased risk of PPCM in women with preexisting hypertension, and this case contributes to the growing body of evidence on the intricate relationship between hypertensive disorders of pregnancy and subsequent cardiac complications.^[3]^

Despite its rarity, PPCM poses significant morbidity and mortality risks, necessitating prompt recognition and intervention. The management of PPCM often involves a delicate balance between optimizing cardiac function and addressing associated risk factors.^[4]^ This case not only underscores the importance of considering hypertensive disorders in the postpartum period but also provides insights into the therapeutic strategies employed in managing PPCM in the context of concurrent hypertension.

## 2. Patient information

Mrs. O.K.O., a 34-year-old female of Nigerian descent, is a typist residing in Awka. She is a Pentecostal Christian and has completed education up to the Senior Secondary Certificate Examination. Mrs. O.K.O. is para 3, with 1 living child and a history of 2 previous pregnancies complicated by preeclampsia.

### 2.1. Chief complaints

Mrs. O.K.O. presented with progressive shortness of breath on exertion over the past 3 months, bilateral leg swelling for the last 10 weeks, and orthopnea for the past month. These symptoms severely impacted her daily activities, including breastfeeding and ambulating short distances.

### 2.2. Medical history

Diagnosed with hypertension 3 years ago during pregnancy, Mrs. O.K.O. occasionally takes Amlodipine 10 mg daily with good adherence. She had a history of preeclampsia in her 2 previous pregnancies. The last delivery was 4 months before the presentation. Mrs. O.K.O. is not known to have diabetes or any known drug allergies.

### 2.3. Family history

Mrs. O.K.O. is the third of 7 siblings in a monogamous family. Her father and elder sister died from an illness of unknown cause. There is no known family history of diabetes, hypertension, sickle cell disease, asthma, or epilepsy.

### 2.4. Psychosocial history

Married to 1 husband, Mrs. O.K.O., and her spouse have 1 living child (daughter). She lives a sedentary lifestyle with a lack of physical activity. There is no history of tobacco or alcohol use. Mrs. O.K.O. has stopped breastfeeding due to worsening symptoms.

### 2.5. Relevant past interventions

Managed for preeclampsia in her 2 previous pregnancies, Mrs. O.K.O. experienced severe hypertension leading to an intrauterine fetal demise at 34 weeks and fetal distress at 36 weeks, necessitating an emergency cesarean section. Unfortunately, the neonate died approximately 6 hours postdelivery.

### 2.6. Current presentation

Mrs. O.K.O.’s symptoms include progressive shortness of breath, bilateral leg swelling, orthopnea, and paroxysmal nocturnal dyspnea. She also reports a dry cough evolving into thick, frothy, whitish sputum. Associated symptoms include early satiety, easy fatigability, and nocturia. Despite herbal medication use, her symptoms worsened, leading to her seeking expert care.

*Note:* The information provided is de-identified to protect patient privacy.

Timeline: Depicting essential milestones related to our diagnoses and interventions

**Table:** 

Timeline of Mrs. O.K.O’s case	Event/intervention	Outcome/response
Before presentation	- Previous pregnancies with preeclampsia	- Intrauterine fetal demise at 34 weeks
	- Previous cesarean section	- Neonatal distress at 36 weeks
3 months before presentation	- Onset of progressive dyspnea on exertion	- Mild impact on sleep and daily activities
2 weeks before presentation	- Initiation of lower extremity swelling, especially feet	- Difficulty wearing shoes, pain in feet
	- Paroxysmal nocturnal dyspnea	- Waking up short of breath after 3 hours of sleep
1 month before presentation	- Development of orthopnea	- Sleeping on a chair due to worsening symptoms
At presentation	- Expert care sought after herbal medication use	- Decompensated heart failure diagnosed
Admission (day 3)	- Initiation of diuretics, spironolactone, and antihypertensives	- Reduction in symptoms and improved exercise tolerance
Day 5	- Introduction of sacubitril/valsartan and digoxin	- Subsiding cough, reduced leg swelling
Day 7 (discharge)	- Comprehensive medication regimen	- Marked improvement in symptoms; patient requesting discharge

*Note:* This timeline visually represents critical events and interventions in Mrs. O.K.O.’s case.

## 3. Diagnostic assessment

### 3.1. Methods

1. Physical examination:
  - Cardiovascular status assessment includes heart sounds, jugular venous pressure, and peripheral pulses.
  - Respiratory examination for signs of heart failure such as crackles and reduced air entry.
  - Abdominal examination for hepatomegaly and ascites.
2. Laboratory testing:
  - Complete blood count reveals anemia (packed cell volume [PCV]: 27%).
  - Serum electrolytes, urea, and creatinine are within normal limits, except for mild elevation in creatinine (1.1 mg/dL).
  - Urinalysis showing proteinuria.
3. Imaging:
  - Chest X-ray demonstrating upper lobe diversion, cardiomegaly, and bilateral pleural effusion.
  - Electrocardiogram revealed sinus tachycardia, left axis deviation, low voltage Q wave, R wave, S wave, and left atrial enlargement.
  - Echocardiography indicated a mildly dilated left atrium, reduced left ventricular ejection fraction (35%), and global wall hypokinesia.

### 3.2. Diagnostic challenges

- Financial constraints may limit access to specific diagnostic modalities.
- Language barriers could hinder effective communication during history-taking and examination.
- Cultural factors may influence patients’ willingness to disclose information or adhere to recommended diagnostic procedures.

### 3.3. Diagnostic reasoning

- Mrs. O.K.O.’s history of hypertension, preeclampsia, and previous pregnancies complicated by heart failure symptoms strongly suggest PPCM.
- The presence of bilateral leg swelling, orthopnea, and paroxysmal nocturnal dyspnea align with heart failure symptoms.
- Anemia contributes to the decompensation of heart failure, evident from the PCV of 27%.

### 3.4. Other diagnoses considered

- Dilated cardiomyopathy secondary to other etiologies.
- Endomyocardial fibrosis.
- Hypertensive heart disease.

### 3.5. Prognostic characteristics

- Staging in heart failure (New York Heart Association [NYHA] classification) was used for prognostic assessment.
- Initial NYHA class III indicated a severe limitation of physical activity.
- Progression to NYHA class II after treatment signifies improving symptoms and functional capacity.

*Note:* The diagnostic assessment involved a comprehensive approach considering clinical, laboratory, and imaging findings, along with addressing potential challenges.

## 4. Therapeutic intervention

### 4.1. Types of intervention

1. Pharmacologic:
  - Diuretics: Intravenous furosemide 40 mg twice daily initiated, later changed to oral torsemide 40 mg daily.
  - Angiotensin receptor-neprilysin inhibitor (ARNI): Sacubitril/valsartan 50 mg twice daily.
  - Digitalis glycoside: Digoxin 0.25 mg daily.
  - Antihypertensive: Telmisartan 80 mg daily (withheld later).
2. Preventive:
  - Anticoagulation: Dabigatran 110 mg daily.
  - Antiplatelet: Clopidogrel 75 mg daily.
  - Statin: Rosuvastatin 20 mg nightly.
3. Self-care:
  - Dietary modification: Restriction of daily salt intake to 2 g/d.
  - Daily weighing: Monitoring for fluid retention.

### 4.2. Administration of intervention

- Intravenous furosemide 40 mg twice daily initially for diuresis, later transitioned to oral torsemide 40 mg daily.
- Sacubitril/valsartan 50 mg twice daily was initiated for ARNI therapy.
- Digoxin 0.25 mg daily is added to enhance myocardial contractility.
- Dabigatran 110 mg daily was started for anticoagulation.
- Clopidogrel 75 mg daily for antiplatelet effect.
- Rosuvastatin 20 mg nightly for lipid management.

### 4.3. Changes in intervention

- Telmisartan withheld:
  - Rationale: ARNI therapy was initiated for its beneficial effects on heart failure. Telmisartan, an angiotensin receptor blocker, was withheld to prevent redundancy in angiotensin II receptor blockade.
- Torsemide adjustment:
  - Rationale: The transition from furosemide to torsemide was made for better bioavailability and efficacy in managing fluid retention.
- Dosage adjustment (day 5):
  - Reduction in IV torsemide to 40 mg daily.
  - Introduction of metoprolol (dose not specified).
  - Rationale: Tailoring the diuretic dosage to the patient’s response. Beta-blocker was introduced for additional heart failure management.

### 4.4. Outcomes

- Marked improvement in symptoms, including reduced dyspnea and leg swelling.
- Transition from NYHA class III to class II, indicating enhanced functional capacity.
- The patient expresses subjective well-being, supporting the effectiveness of the therapeutic interventions.

*Note:* The adjustments in the intervention were made based on the patient’s response and the evolving clinical picture.

## 5. Follow-up and outcomes

### 5.1. Clinician-assessed outcomes

- Day 5 (Registrer’s ward round [RWR]):
  - Reduced symptoms of cough, leg swelling, and orthopnea.
  - Objective improvement in vital signs (pulse rate: 98 b/m, BP: 140/90 mm Hg).
  - The cough subsided, leg swelling markedly reduced, and orthopnea improved.
- Day 7 (RWR-discharge):
  - Significant improvement in breathlessness and leg swelling.
  - The patient is happier, not pale, with pedal edema up to the distal 1/3 of the tibia.
  - Pulse rate: 87 b/m, NVR, BP: 142/89 mm Hg.

### 5.2. Patient-assessed outcomes

- Day 5 (RWR):
  - Subjective improvement in cough, leg swelling, and ability to sleep supine.
  - The patient reports reduced overall discomfort and improved exercise tolerance.
- Day 7 (RWR-discharge):
  - The patient expresses marked improvement in breathlessness and leg swelling.
  - No new complaints were reported, indicating overall satisfaction with the treatment.

### 5.3. Follow-up test results

- Day 3:
  - Chest X-ray, electrocardiogram, echo, abdominal pelvic ultrasound scan, PCV, urinalysis, and serum electrolytes, urea, and creatinine were performed.
  - Results indicated cardiomegaly, pleural effusion, reduced left ventricular ejection fraction, hepatomegaly, anemia, and proteinuria.

### 5.4. Intervention adherence and tolerability

- Adherence is assessed through daily nursing care and discussions with the patient.
- Tolerability is assessed based on the absence of adverse effects and symptom improvement.

### 5.5. Adverse and unanticipated events

- No adverse events were reported during the follow-up period.
- The patient exhibited good tolerance to the prescribed medications.

### 5.6. Conclusion

Mrs. O.K.O. responded positively to the therapeutic interventions, significantly improving clinician- and patient-assessed outcomes. Adherence was monitored through nursing care, and the absence of adverse events suggests tolerability. Regular follow-up remains crucial to assess the long-term efficacy and compliance with the prescribed regimen.

*Note:* The follow-up and outcomes assessment involved a combination of objective clinical measures, patient-reported experiences, and ongoing monitoring of test results.

## 6. Discussion

### 6.1. Strengths in management

1. Multidisciplinary approach:
  - Involvement of various specialists in managing heart failure, hypertension, and obstetric history.
  - Collaborative decision-making contributed to a comprehensive treatment plan.
2. Tailored medication regimen:
  - Individualized pharmacologic interventions addressing heart failure, hypertension, and associated risk factors.
  - Dynamic adjustments based on the patient’s response enhanced treatment efficacy.
3. Comprehensive follow-up:
  - Regular clinical assessments, including physical examinations and vital sign monitoring.
  - Follow-up test results provided insights into the evolving clinical picture.

### 6.2. Limitations in management

1. Diagnostic challenges:
  - Financial, language, and cultural barriers may have influenced the diagnostic process.
  - Limited access to specific diagnostic modalities could impact the thoroughness of the assessment.
2. Patient adherence:
  - Adherence to prescribed lifestyle modifications, such as salt restriction, might be challenging.
  - The patient’s ability to sustain long-term adherence to the complex medication regimen is still being determined.

### 6.3. Relevant medical literature

- The Heart Failure Association of the European Society of Cardiology emphasizes the importance of early diagnosis and a multidisciplinary approach in managing PPCM.^[1]^
- Studies highlight the increased risk of heart failure in women with a history of hypertensive disorders in pregnancy, aligning with Mrs. O.K.O.’s case.^[2]^
- The efficacy of sacubitril/valsartan in heart failure with reduced ejection fraction is well-documented, supporting its inclusion in Mrs. O.K.O.’s treatment plan.^[3]^

### 6.4. The rationale for conclusions

- Mrs. O.K.O.’s positive response to treatment aligns with established literature on the management of PPCM and heart failure.
- Regular follow-up assessments and appropriate intervention adjustments contributed to improved symptoms and functional capacity.

### 6.5. Takeaway lessons

1. Early recognition matters:
  - Awareness of cardiovascular risks in postpartum women, especially those with a history of hypertensive disorders, is crucial for early diagnosis and intervention.
2. Tailoring treatment is essential:
  - Individualized management, considering patient-specific factors, contributes to positive outcomes.
3. Long-term adherence challenges:
  - The case highlights potential challenges sustaining long-term adherence to complex medication regimens and lifestyle modifications.

### 6.6. Conclusion

This case underscores the importance of a multidisciplinary approach, individualized treatment plans, and ongoing follow-up in managing complex cardiovascular conditions in postpartum women. While recognizing strengths in management, it also emphasizes the challenges and uncertainties associated with long-term adherence and cultural influences.

## 7. Patient perspective

Mrs. O.K.O. reflects on the treatments received with a mix of gratitude and challenges. She expresses relief in alleviating her distressing symptoms, notably improved breathing and reduced leg swelling. The medications prescribed, including diuretics and heart failure medications, have played a pivotal role in restoring her ability to perform daily activities. Mrs. O.K.O. appreciates the collaborative approach of the healthcare team, emphasizing the importance of the expert care she received.

However, she acknowledges the complexity of the prescribed medications and the challenge of adhering to the recommended lifestyle changes. The multitude of pills and the dietary restrictions pose ongoing challenges, and she is aware of the importance of long-term adherence for sustained well-being. Mrs. O.K.O. expresses gratitude for the supportive care but recognizes the journey ahead, understanding the need for ongoing monitoring and adjustments in her treatment plan.

## Acknowledgments

The authors thank Mrs. O.K.O. for her participation and permission to share her clinical details for educational purposes.

## Author contributions

**Conceptualization:** Chukwuka Elendu.

**Data curation:** Chukwuka Elendu, Osinachi K. Okoye.

**Formal analysis:** Chukwuka Elendu, Osinachi K. Okoye.

**Funding acquisition:** Chukwuka Elendu, Osinachi K. Okoye.

**Investigation:** Chukwuka Elendu.

**Methodology:** Chukwuka Elendu, Osinachi K. Okoye.

**Project administration:** Chukwuka Elendu, Osinachi K. Okoye.

**Resources:** Chukwuka Elendu, Osinachi K. Okoye.

**Software:** Chukwuka Elendu, Osinachi K. Okoye.

**Supervision:** Chukwuka Elendu, Osinachi K. Okoye.

**Validation:** Chukwuka Elendu, Osinachi K. Okoye.

**Visualization:** Chukwuka Elendu, Osinachi K. Okoye.

**Writing—original draft:** Chukwuka Elendu.

**Writing—review & editing:** Chukwuka Elendu.
